# Regimen simplification and medication adherence: Fixed-dose versus loose-dose combination therapy for type 2 diabetes

**DOI:** 10.1371/journal.pone.0250993

**Published:** 2021-05-04

**Authors:** Anna-Katharina Böhm, Udo Schneider, Jens Aberle, Tom Stargardt

**Affiliations:** 1 Hamburg Center for Health Economics, University of Hamburg, Hamburg, Germany; 2 Techniker Krankenkasse, Hamburg, Germany; 3 Department Endocrinology and Diabetology, University Obesity Center Hamburg, University Hospital Hamburg-Eppendorf, Hamburg, Germany; Universidad Miguel Hernandez de Elche, SPAIN

## Abstract

**Background:**

Suboptimal patient adherence to pharmacological therapy of type 2 diabetes may be due in part to pill burden. One way to reduce pill burden in patients who need multiple medications is to use fixed-dose combinations. Our study aimed to compare the effects of fixed-dose combination versus loose-dose combination therapy on medication adherence and persistence, health care utilization, therapeutic safety, morbidities, and treatment modification in patients with type 2 diabetes over three years.

**Methods:**

Using administrative data, we conducted a retrospective controlled cohort study comparing type 2 diabetes patients who switched from monotherapy to either a fixed-dose combination or a loose-dose combination. Adherence was assessed as the primary endpoint and calculated as the proportion of days covered with medication. After using entropy balancing to eliminate differences in observable baseline characteristics between the two groups, we applied difference-in-difference estimators for each outcome to account for time-invariant unobservable heterogeneity.

**Results:**

Of the 990 type 2 diabetes patients included in our analysis, 756 were taking a fixed-dose combination and 234 were taking a loose-dose combination. We observed a statistically significantly higher change in adherence (year one: 0.22, p<0.001, year two: 0.25, p<0.001, and year three: 0.29, p<0.001) as well as higher persistence and a smaller change in the number of drug prescriptions in each of the three years in the fixed-dose combination group compared to the loose-dose combination group. The differences were most pronounced in patients who were poorly adherent, had a high pill burden, or did not have a severe concomitant disease.

**Conclusion:**

Our results indicate that taking a fixed-dose combination can lead to a significant improvement in adherence to pharmacological therapy of type 2 diabetes compared to a loose-dose combination. In particular, these findings suggest that reducing pill burden may improve disease management among patients with more complex medication demand and patients who have demonstrated poor medication adherence.

## Introduction

Despite the availability of effective treatments, at least 45% of patients with type 2 diabetes (T2D) fail to achieve glycaemic control, resulting in unnecessarily high rates of morbidity and mortality [1]. One of the main contributors to adequate glycaemic control is medication adherence [2]. However, previous studies have reported that adherence to antidiabetic medications is generally poor, often not surpassing the conventional threshold of 80% [1]. One reason for poor adherence may be the complexity of medication regimens and pill burden in T2D therapy: in cases where lifestyle modifications (diet and exercise) are no longer sufficiently effective, treatment guidelines generally recommend oral antidiabetic medication [3]. Treatment usually begins with monotherapy but may progress to dual or triple therapy, with each medication having a different mechanism of action to achieve additive or synergistic effects. Ultimately, injectable treatment with insulin may become necessary to achieve adequate glycaemic control [3].

One way to make pharmacological treatment simpler for patients who need multiple medications is to use fixed-dose combinations (FDCs). FDCs contain two or more active components in a single dosage form, reducing pill burden and thereby potentially facilitating adherence to treatment [4]. At the same time, however, FDCs lack flexibility because they do not allow physicians to titrate doses individually or easily identify the substance or substances responsible for suboptimal efficacy or adverse events [5]. Lastly, it is conceivable that the simplicity of treatment with FDCs may minimize patients’ awareness of the progressed severity of the disease. In short, it remains unclear whether FDCs provide greater health benefits overall than the corresponding loose-dose combinations (LDCs).

For the most part, the previous literature has reported positive effects on adherence and various secondary outcomes for FDCs compared to LDCs in the treatment of T2D [6, 7]. However, there are three notable weaknesses in the studies on this subject that have been published to date. First, establishing causality is difficult. Although studies based on data from randomized controlled trials are usually considered as the gold standard of evidence, they cannot reflect outcomes under everyday conditions [8]. However, T2D patients are often not motivated to change their lifestyle or misperceive their behavior, which could increase the need for studies based on real world data [9]. In addition, differences in adherence have been identified as the main reason for the gap between clinical efficacy in randomized controlled trials and effectiveness in the real world use of T2D medication [10]. Observational studies also have weaknesses though [8]. Chief among these is their inability to account for non-randomized drug assignment, which can lead to biased estimates due to selection or omitted variable bias. In our study, we attempt to overcome both weaknesses by employing an advanced two-step risk-adjustment procedure, combining entropy balancing (EB) with difference-in-difference (DiD) estimation to create a quasi-experimental setting using administrative data. Another characteristic of the studies published to date is that they have focused mainly on the US health care market [11–18]. With one exception from Greece [19], there is little evidence on outcomes in European health care markets, which differ considerably from the US health care sector, e.g., with respect to drug reimbursement policies that may affect medication adherence. A further weakness of most of the previous studies is their short follow-up period of no more than 12 months [11–17, 19, 20]. Literature on medication compliance suggests that long-term compliance is more difficult to obtain than short-term compliance and requires a combination of interventions to be effective [21]. Our paper contributes to the literature by extending the observation period to three years, which may provide important insights into the sustainability of differences in adherence and allows lagged exposure time to medication to be investigated. Moreover, to our knowledge only two real world data studies have investigated the effect of antidiabetic FDCs on health care utilization, and no such study has analyzed therapeutic safety, disease-related morbidities or therapy modification [13, 18]. In our study, we address these gaps in the literature by examining the example patients who added sitagliptin (a Dipeptidyl peptidase 4 inhibitor) to metformin, which together represent the most widely prescribed FDC in our data set.

## Methods

The present study is a retrospective, observational, non-interventional study and all data were fully anonymized, therefore approval by an Ethics Committee was not required.

### Study design and sample

To estimate the effect of replacing monotherapy with either an FDC or an LDC for the treatment of T2D, we undertook a retrospective controlled (i.e., including a control group) cohort study. We used administrative data from the Techniker Krankenkasse, the largest statutory health insurer in Germany. Approximately 90% of the population in Germany is covered by about 100 different statutory health insurers, and Techniker Krankenkasse provides coverage for 14.5% of these individuals. Almost all of the remaining population is covered by fully substitutive private health insurance. Our data set included longitudinal patient-level information on sociodemographic status, in- and outpatient medical diagnoses and services, and pharmaceutical prescriptions between 2013 and 2017. We use the term outpatient to describe office-based solo and group practices, which in the German health care system are the setting in which almost all primary care and the vast majority of specialist care are provided.

**Fig 1:**
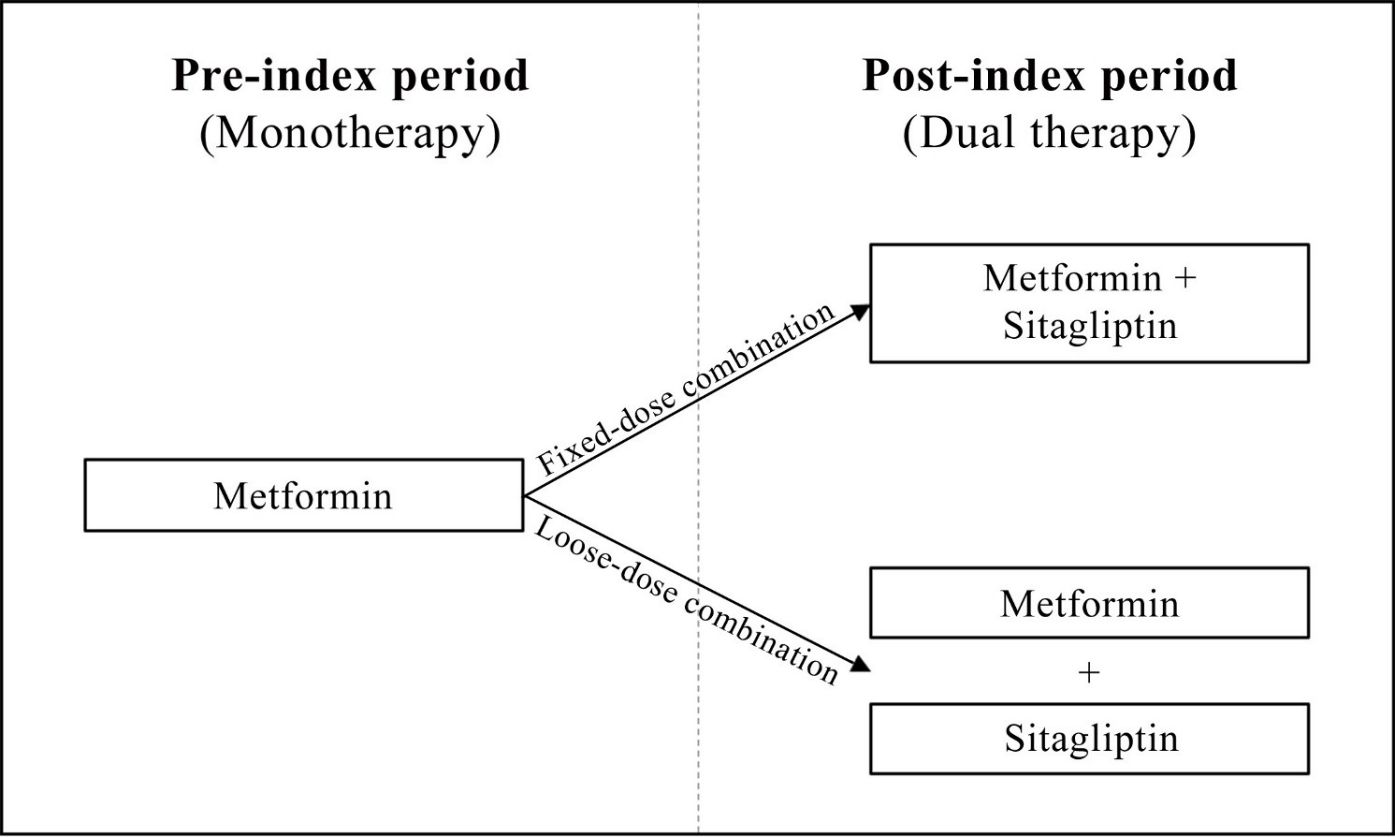
Selection of study cohorts. All patients received metformin monotherapy in a pre-index period of one year and added sitagliptin either as a fixed-dose combination or loose dose combination in a post-index period of three years.

We constructed two mutually exclusive cohorts of T2D patients who had added sitagliptin to metformin monotherapy and continued to take this combination over a period of three years, either as an FDC or an LDC (see Fig 1). We defined T2D patients as those who had received an International Classification of Diseases (ICD) diagnosis of T2D (E11) in two or more outpatient claims in four preceding quarters or in one or more hospital claims in a one-year baseline period. We further restricted the study population to patients who, during the study period, (i) had continuous health insurance coverage with Techniker Krankenkasse, (ii) were not pregnant, and (iii) received no antidiabetic prescription other than for metformin and sitagliptin. In order to maximize comparability between the cohorts, we excluded individuals who switched between an FDC and an LDC during the study period. Additionally, patients had to have taken monotherapy for a sufficient amount of time (i.e., the first observable prescription of metformin had to be no later than 180 days before adding sitagliptin). In accordance with the previous literature, we defined LDC therapy as co-administration of metformin and sitagliptin with at least two overlap periods of 15 days or more, whereas we identified FDC therapy directly by means of its unique Anatomical Therapeutic Chemical Classification (ATC) code (A10BD07) [11, 12]. The index date was defined as the first prescription fill for the additive substance (LDC) or the first prescription fill for the FDC, in 2014. We measured baseline characteristics for determining patient-level risk profiles over a period of one year prior to the index date and assessed outcomes for each of the three years of the follow-up period.

### Study outcomes

The main outcome of interest was medication adherence, measured as the proportion of days covered (PDC) with medication. The previous literature applies a variety of methods to estimate patients’ adherence when using retrospective data. We based our choice of adherence measure on the official recommendations for chronic diseases published by the US Pharmacy Quality Alliance [22]. Secondary outcomes were persistence to treatment, health care utilization, therapeutic safety, morbidities, and treatment modification. We assessed outcomes annually up to three years after the index date. If necessary, we corrected for prescription fills ranging from one year to the next, allowing for takeover of medication.

#### Adherence

We calculated the PDC as the ratio of the number of days covered by the prescription fills (here: defined daily doses) divided by the number of days in the specified time interval (here: 365 days for each year):

$$PDC=\left(\frac{Number\;of\;days\;in\;period\;“covered”}{Number\;of\;days\;in\;period}\right)\times 100\%$$

In contrast to the medication possession ratio, which is a more traditional measure of adherence, the numerator of the PDC is not a simple summation of the days’ supply. The PDC adds information through the use of indicator variables flagging medication coverage for each day, based on prescription fill dates and days’ supply. This is advantageous when assessing adherence to multiple medications, such as LDCs. Following the recommendation of the Medication Adherence and Persistence Special Interest Group of the International Society for Pharmacoeconomics and Outcomes Research (ISPOR), we constructed the PDC for LDC therapy using only days in the numerator when both prescribed medications were available [23, 24]. If the medications were refilled before the end of supply, the fill date was shifted forward to the day after the end of the supply of the previous filled prescription. Therefore, we truncated PDCs greater than 100% at 100%. We adopted the computational calculation from Leslie (2007) and Leslie et al. (2008) [25, 26].

#### Persistence

We defined medication persistence as the duration of time from initiation to discontinuation of therapy, measured as days from the date of the first dual therapy to the date of the first prescription gap longer than 30 consecutive days. The length of the permissible gap was chosen based on previous persistence studies in T2D patients [13]. Patients taking an LDC were considered non-persistent if the permissible gap was exceeded for either of the two substances. We used Kaplan-Meier curves with time to discontinuation to display the percentage of FDC vs. LDC patients remaining on therapy in the post-index period. If the first prescription gap started within the last 30 days of the three years follow-up period, we assumed discontinuation of treatment (i.e., non-persistence).

#### Health care utilization

We determined the number of outpatient cases (comprising both specialist and general practitioner visits), the number of T2D-related outpatient cases, the number of total and T2D-related pharmaceutical prescriptions, the proportion of individuals with at least one emergency visit, and the proportion of individuals with at least one emergency visit due to T2D-related morbidities, such as myocardial infarction (see next section). Each outcome was assessed annually up to three years after the index date.

#### Therapeutic safety

We assessed therapeutic safety based on the occurrence of one or more adverse drug events, which we identified using ICD-10 diagnoses following a list recently published in the official journal of the German Medical Association and the German National Association of Statutory Health Insurance Physicians [27]. The list contains the ten most frequent main and secondary diagnoses associated with adverse drug events in hospitals in Germany (for detailed information, see S1 Table). For each year, we measured the share of individuals with at least one adverse event.

#### Morbidity outcomes

We compared the prevalence of disease-related morbidities between the FDC and the LDC groups. These included microangiopathic complications (eye complications, renal failure, diabetic foot syndrome, and peripheral neuropathy) as well as macroangiopathic complications (angina pectoris, myocardial infarction, ischemic heart disease, heart failure, and cerebrovascular diseases) [28]. We considered morbidities to be relevant only if the respective ICD-10 diagnosis (for detailed information, see S2 Table) was confirmed at least once in inpatient settings or at least twice in outpatient settings during each year.

#### Treatment modification

In addition to our main study cohort, we identified individuals who switched from monotherapy to FDC or LDC dual therapy but had to modify the latter by (i) adding or switching to another oral antidiabetic drug (ATC code A10B) or (ii) adding insulins (ATC code A10A) after 180 days or later (i.e., ignoring inclusion criterion (iii) described in section “Study design and sample” after 180 days after the index date). We measured the share of individuals that modified treatment at any point prior to or during each of the three years of the follow-up period.

### Statistical analysis and risk-adjustment

In order to reduce confounding, we applied a two-step risk adjustment combining (i) weighting based on EB to control for observable baseline characteristics between cohorts and (ii) conditional DiD estimation to control for time-invariant unobservable heterogeneity between cohorts. We measured baseline characteristics over a period of one year prior to the index date and assessed outcomes for each of the three years of the follow-up period.

First, we implemented EB as a reweighting technique to maximize similarity in the mean and variance of a set of predefined conditioning variables between the two cohorts, thus creating a synthetic control group [29]. To achieve covariate balance, EB recalibrates weights to each control individual such that the pre-specified balancing requirements are satisfied. The weights obtained in the reweighting step can then be used as sampling weights in the regression step. Based on adherence literature, we included a set of conditioning variables that are considered to have a high predictive potential for treatment assignment and the outcomes: (i) sociodemographic variables (age, gender, and insurance category as a proxy for socioeconomic background), (ii) generic comorbidity measures (29 of 31 Elixhauser groups (i.e., except for diabetes) and 31 of 32 pharmacy-based metrics (i.e., except for diabetes)), (iii) indicators for T2D-specific comorbidities (compare section “Morbidity outcomes”; if comorbidities were assessed as outcomes, they have been excluded as conditioning variables), (iv) adherence reported as the PDC, (v) an indicator for major polypharmacy (i.e., the prescription of >5 distinct ATC codes), (vi) an indicator for whether patients had enrolled in a disease management program (DMP) for T2D (which could e.g., reflect the patient’s health literacy or quality of communication between the patient and health care provider), and (vii) an indicator for whether physicians treated any patients (either in or outside of the study population) who had enrolled in a T2D DMP [30–33]. If physicians treated (any) patients who had enrolled in a DMP, this may indicate that physicians were adhering to latest treatment guidelines. We included binary variables to determine whether a patient had at least one of the specified inpatient diagnoses and prescriptions or two of the specified outpatient diagnoses in the pre-index period. In order to evaluate the balancing-performance, we calculated standardized mean differences to compare the covariate balance before and after weighting.

The second step was the regression, where we eliminated differences in changes in outcomes between the FDC and the LDC groups due to time-constant unobserved factors after DiD estimation for each year. The DiD estimator measured whether there was a differential change in outcomes before and after adding sitagliptin to metformin, i.e., between the pre- and post-index period in the FDC group relative to the LDC group. Our empirical strategy relied on the assumption that, in absence of combination products (e.g., if they were not available), patients would have switched to an analogous LDC. This means that, in the absence of treatment the unobserved differences between treatment and control groups would be the same over time. We estimated three weighted least squares (WLS) regressions (one for each year in the post-index period), using the weights generated in the EB step. The structural equation of interest can be described as follows:

$$Y_{it}=\beta_{0}+\beta_{1}Treat_{it}+\beta_{2}Post_{t}+\beta_{3}Treat_{it}*Post_{t}+X_{i}+\varepsilon_{it}$$ (1)

where Y_it_ refers to the outcomes of interest for individual i in time period t. Treat_it_ is a dummy variable that indicates if individual i belonged to the FDC cohort, and Post_t_ is a dummy variable that takes the value one in the post-index period. Therefore, the interaction term Treat_it_ ⚹ Post_t_ takes the value one for individuals who added sitagliptin in an FDC and were observed in the post period. Hence, β_3_ yields the DiD estimator, indicating the expected mean differences in changes in the outcomes due to treatment choice in the respective post period (i.e., the isolation of the average treatment effect on the treated (ATT)). We added the vector Xi that contains the set of conditioning variables form the balancing step in order to reduce variance in the outcomes and to increase the precision of the estimates. To account for heteroscedasticity, robust standard errors were used.

The effect on the binary variables (emergency visit, therapeutic safety, and morbidities) may be interpreted as changes in the linear probability Pr(Outcome_it_ = 1). A drawback of linear probability models is that they may predict probabilities outside the interval [0,1]. However, this may be neglected as we are only interested in the DiD coefficient.

The parallel trend assumption was satisfied by plotting the relevant outcomes for each quarter of the 12-month baseline period (for detailed information, see S1 Fig). The plots show that the outcomes of the LDC cohort display how outcomes for the FDC cohort would have developed in the absence of FDC therapy. Therefore, the LDC cohort is an appropriate control group in our study.

For the secondary outcomes that can be observed only in the post-index period (i.e., therapy modification and persistence), we applied a simple WLS regression. The regression analysis considering persistence was merely based on the first year of the post-index period. We used the data of the beginning of the second year of the post-index period to obtain the full lengths of the prescription gap if the first prescription gap began within 30 days of the end of the first year of the post-index period.

Entropy balancing was performed using the package ebal for R, version 3.6.1, and regression analyses were performed using SAS 9.4M4_V2. P-values less than. 10 were regarded as statistically significant in our analyses.

### Subgroup analyses

In order to explore differences between particular groups of patients, we conducted subgroup analyses (i) for individuals who had already been highly adherent (PDC>0.8) in the pre-index period in order to assess whether the effect would be different for more or less adherent individuals, (ii) for individuals with major polypharmacy (i.e., the prescription of >5 distinct ATC codes in the last quarter before the index date), because additional complexity in medication regimens might affect selection into treatment and medication adherence, and (iii) excluding individuals who had either psychological disorders (ICD F) or cancer (ICD C), because the T2D therapy might be considered to be of minor priority in such cases [34]. We assigned new EB weights to the subsets of individuals in the control groups.

### Sensitivity analyses

To investigate the robustness of our findings, we conducted two types of sensitivity analyses. First, given that there is no gold standard to estimate adherence to multiple medications in administrative data, we calculated the PDC of the LDC cohort as the mean PDC of both substances, rather than only taking account of the days during which both substances were available. We subsequently repeated the analysis of adherence as described above. Second, patients with pre-existing severe renal failure may be more prone to receive LDC therapy because of necessary dose adjustment. Hence, we repeated our analyses, formally excluding the Elixhauser group 14 (renal failure).

## Results

### Descriptives and balancing

Applying the inclusion and exclusion criteria yielded a data set with a total of 990 individuals of whom 756 were in the FDC and 234 in the LDC group. EB created a highly balanced distribution of all baseline characteristics, although observable differences prior to weighting were already small, indicating that the control group was highly suitable (see Table 1 or, for more detail, S3 Table).

**Table 1:** Baseline characteristics of fixed-dose and loose-dose combination cohorts before and after entropy balancing.

Variables^a^	FDC	LDC		SMD	
		Before EB	After EB	Before EB	After EB
Sample size (N)	756	234			
Mean age (years)	62.10	63.08	62.17	-0.09	-0.01
Male	71.69	68.38	71.78	0.07	0.00
Insurance status^b^:					
voluntary	15.08	13.68	15.06	0.04	0.00
T2D DMP					
DMP enrolment patient	70.77	73.50	70.84	-0.06	0.00
DMP supporting physician	90.74	88.89	90.75	0.06	0.00
Adherence	69.25	73.95	69.31	-0.20	0.00
Major polypharmacy	69.31	72.22	69.41	-0.06	0.00
Elixhauser comorbidities (for more details see S3 Table)					
before EB	9 of 29 |SMD| > 0.10				
after EB	0 of 29 with |SMD| > 0.00				
T2D-specific comorbidities (for more details see S3 Table)					
before EB	1 of 7 with |SMD| > 0.10				
after EB	0 of 7 with |SMD| > 0.00				
Pharmacy-based classes (for more details see S3 Table)					
before EB	4 of 31 with |SMD| > 0.10				
after EB	0 of 31 with |SMD| > 0.00				

FDC: Fixed-dose combination, LDC: Loose-dose combination, SMD: Standardized mean difference, EB: Entropy balancing, T2D: Type 2 diabetes mellitus, DMP: Disease management program

^a^All values in % unless indicated otherwise.

^b^Insurance category refers to individuals’ enrolment in statutory health insurance as being mandatory, which is the case below a certain income threshold, or voluntary, which is the case above this threshold. Put simply, for historical reasons, higher-income individuals in Germany may choose between statutory health insurance and fully substitutive private health insurance.

### Main results

Table 2 shows the results for the DiD estimator, which measures the ATT, and its standard error for FDC vs. LDC treatment in the three years after patients added sitagliptin to metformin (for the (adjusted) mean outcomes, see S4 Table).

**Table 2:** Difference-in-difference regression results.

	Difference-in-difference estimators					
Outcomes	Year 1		Year 2		Year 3	
	ATT	SE	ATT	SE	ATT	SE
*Adherence and Persistence*						
Proportion of days covered	0.22***	0.02	0.25***	0.02	0.29***	0.03
Days until discontinuation^a^	119.04***	8.31	N/A	N/A	N/A	N/A
*Indicators for health care utilization*						
Outpatient cases	-0.16	0.63	0.54	0.63	0.52	0.63
due to diabetes	-0.07	0.28	0.17	0.29	-0.02	0.29
Pharmaceutical prescriptions	-4.02***	1.07	-3.48***	1.14	-4.87***	1.77
due to diabetes	-4.59***	0.20	-3.66***	0.27	-3.27***	0.23
Proportion with emergency visits	0.02	0.04	0.03	0.04	0.03	0.04
due to T2Dcomorbidities	0.01	0.02	-0.01	0.02	0.01	0.02
*Therapeutic safety*						
Proportion with adverse drug events	0.04	0.03	0.03	0.03	0.01	0.03
*Comorbidities*						
Proportion with microangiopathic complications						
Eye complication	0.00	0.04	-0.03	0.04	-0.07	0.05
Renal failure	0.00	0.03	0.01	0.04	-0.02	0.04
Diabetic foot syndrome/ Periphere neuropathy	0.02	0.04	0.03	0.04	-0.02	0.05
Proportion with macroangiopathic complications						
Myocardial infarction	0.00	0.02	0.02	0.02	0.02	0.02
Ischemic heart disease	0.00	0.03	-0.02	0.03	-0.01	0.04
Angina pectoris	0.00	0.02	0.01	0.02	-0.01	0.03
Heart failure	0.01	0.01	0.01	0.02	0.02	0.02
Cerebrovascular disease	0.00	0.02	0.00	0.02	-0.01	0.03
*Treatment modification*^*b*^						
Proportion with alternative						
Oral antidiabetics	0.00	0.03	0.02	0.04	-0.01	0.03
Insulins	0.03**	0.01	0.03	0.03	-0.03	0.04

ATT: Average treatment effect on the treated represents excess outcomes attributable to FDC with

* p<0.10,

** p<0.05,

***p<0.01, SE: Standard error

^a^refers to discontinuation of treatment in year one of post-index period, estimated using weighted least squares.

^b^based on individuals who modified their dual theapy (N: FDC = 732, LDC = 229)

#### Adherence and persistence

**Fig 2:**
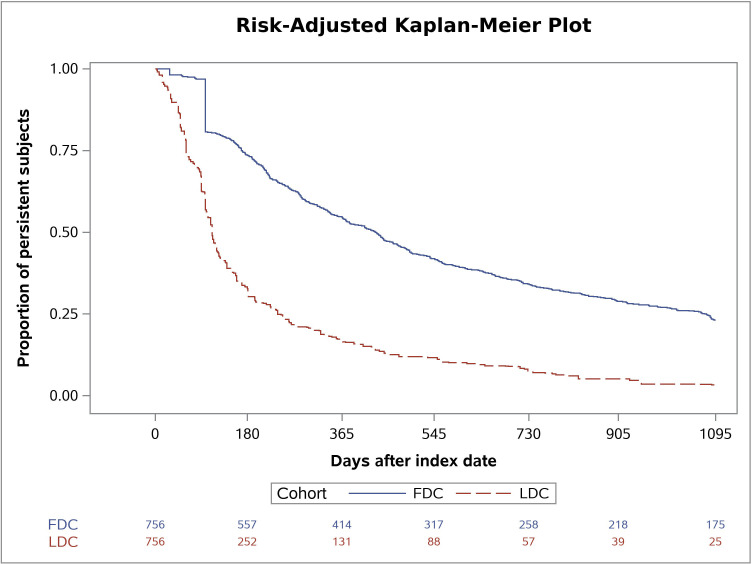
Risk-adjusted Kaplan-Meier plot. Weighted Kaplan-Meier curves displaying the proportion of individuals without discontinuation of treatment in the fixed-dose combination (FDC) and loose-dose combination (LDC) cohort.

The change in adherence to dual therapy was statistically significantly greater in patients receiving an FDC compared to the change experienced by the LDC group in each of the three years of the post-index period. It was largest in the third year (0.22, p<0.001; 0.25, p<0.001; 0.29, p<0.001). In line with this, the number of days without a therapy gap of 30 or more days in the first year of the post-index period was statistically significantly higher in the FDC group (119.04, p<0.001). The risk-adjusted Kaplan-Meier curves displaying the proportion of persistent individuals over three years are shown in Fig 2.

#### Health care utilization

We did not observe any statistically significant different changes in either the number of outpatient cases or the number of outpatient cases due to T2D between the two groups in any of the three years. As expected, the change in the total number of pharmaceutical prescriptions was statistically significantly smaller for the FDC cohort (-4.02, p<0.001; -3.48, p<0.001, -4.87, p<0.001). This effect was strongly driven by the antidiabetic therapy (-4.59, p<0.001; -3.66, p<0.001; -3.27, p<0.001). Furthermore, we found no statistically significant differences in changes in the proportion of individuals with emergency visits between the two groups.

#### Therapeutic safety

FDC therapy did not lead to a statistically significant different change in the proportion of individuals with adverse drug events compared to LDC therapy.

#### Morbidity outcomes

The changes in the shares of patients with T2D-specific morbidities did not differ statistically significantly between the two groups in all three years.

#### Treatment modification

We identified patients who modified their dual therapy (e.g., by switching to another combination, adding another oral antidiabetic medication, or taking insulin). This population consisted of 732 patients taking an FDC and 229 taking an LDC for at least 180 days before treatment modification. We observed no statistically significant differences between the two groups with regard to the proportion of individuals adding or switching to other oral antidiabetics. For individuals who started dual therapy by taking an FDC, we observed a statistically significant effect towards adding insulin in year one after the index date (0.03, p = 0.03), but no differences in the later years.

### Subgroup analyses

Table 3 displays the results of the three subgroup analyses. Although the ATT for adherence was slightly smaller when we considered only the subgroup of individuals who had been highly adherent at baseline compared to all patients, the estimator remained positive and highly statistically significant (0.17, p<0.001; 0.22, p<0.001; 0.25, p<0.001). Our results with respect to the other outcomes remained stable.

**Table 3:** Difference-in-difference regression results for subgroups.

	Highly adherent individuals			Individuals with polypharmacy			Excluding individuals with ICD F/ C		
	(N: FDC = 319, LDC = 114)			(N: FDC = 235, LDC = 85)			(N: FDC = 374, LDC = 108)		
Outcomes^a^	Year 1	Year 2	Year 3	Year 1	Year 2	Year 3	Year 1	Year 2	Year 3
*Adherence and Persistence*									
Proportion of days covered	0.17(0.02)***	0.22(0.03)***	0.25(0.04)***	0.20(0.03)***	0.30(0.04)***	0.34(0.05)***	0.22(0.03)***	0.29(0.04)***	0.30(0.05)***
Days until discontinuation^b^	101.12(12.41)***	N/A	N/A	101.28(13.31)***	N/A	N/A	118.10(14.15)***	N/A	N/A
*Indicators for health care utilization*									
Outpatient cases	-0.30(0.84)	0.27(0.78)	-0.18(0.79)	-0.46(1.12)	0.32(1.00)	-0.48(1.25)	-0.65(0.92)	0.16(0.75)	0.11(1.02)
due to diabetes	-0.25(0.40)	0.08(0.40)	0.12(0.44)	-0.47(0.64)	-0.03(0.64)	0.03(0.59)	0.30(0.38)	0.43(0.40)	0.18(0.40)
Pharmaceutical prescriptions	-4.05(1.31) ***	-4.16(1.34) ***	-3.67(1.39) ***	-1.42(1.82)	0.06(1.98)	-1.79(2.12)	-4.50(1.41) ***	-3.00(1.44) **	-2.57(1.42) *
due to diabetes	-4.44(0.26) ***	-3.78(0.26) ***	-3.67(0.29) ***	-4.44(0.34) ***	-3.16(0.41) ***	-2.91(0.48) ***	-4.48(0.35) ***	-3.27(0.38) ***	-2.99(0.40) ***
Proportion with emergency visits	0.00(0.05)	0.05(0.05)	0.05(0.05)	0.00(0.06)	-0.11(0.07)	-0.14(0.08) *	0.07(0.06)	0.07(0.06)	0.03(0.07)
due to T2D comorbidities	0.01(0.03)	0.00(0.03)	0.02(0.03)	0.00(0.03)	-0.05(0.05)	-0.02(0.05)	0.03(0.02)	-0.02(0.04)	-0.02(0.05)
*Therapeutic safety*									
Proportion with adverse drug events	0.02(0.05)	0.01(0.05)	-0.01(0.05)	0.02(0.04)	-0.09(0.07)	-0.06(0.06)	-0.01(0.05)	-0.02(0.05)	0.00(0.05)
*Comorbidities*									
*Proportion with microangiopathic complications*									
Eye complication	0.00(0.05)	-0.03(0.06)	-0.05(0.06)	0.02(0.05)	-0.10(0.07)	-0.06(0.06)	0.00(0.06)	-0.08(0.08)	-0.06(0.08)
Renal failure	-0.02(0.05)	0.02(0.06)	0.00(0.06)	-0.02(0.05)	-0.19(0.09) **	-0.17(0.09) **	0.02(0.05)	0.06(0.06)	-0.00(0.06)
Diabetic foot syndrome/ Periphere neuropathy	0.00(0.06)	0.04(0.06)	0.05(0.06)	0.02(0.07)	0.05(0.08)	0.06(0.08)	0.00(0.04)	0.00(0.06)	-0.09(0.06)
*Proportion with macroangiopathic complications*									
Myocardial infarction	0.03(0.03)	0.01(0.03)	0.01(0.03)	0.01(0.04)	0.00(0.06)	0.05(0.04)	0.01(0.02)	0.01(0.03)	0.00(0.02)
Ischemic heart disease	-0.01(0.04)	-0.06(0.04)	-0.04(0.04)	0.02(0.06)	-0.04(0.07)	0.00(0.07)	0.00(0.06)	-0.05(0.06)	-0.04(0.07)
Angina pectoris	-0.01(0.03)	0.01(0.02)	0.00(0.03)	-0.03(0.03)	-0.01(0.03)	-0.07(0.05)	-0.01(0.03)	0.00(0.03)	-0.08(0.05)
Heart failure	0.03(0.02)	-0.02(0.04)	0.02(0.04)	0.01(0.03)	-0.06(0.06)	-0.06(0.06)	0.01(0.01)	0.00(0.03)	0.03(0.02) *
Cerebrovascular disease	0.01(0.03)	0.01(0.03)	0.00(0.03)	0.00(0.04)	-0.01(0.05)	-0.05(0.06)	0.01(0.02)	0.02(0.02)	0.02(0.02)

FDC: Fixed-dose combination, LDC: Loose-dose combination, ICD: International classification of diseases

^a^Values show ATT(SE): Average treatment effect on the treated (standard error) with

* p<0.10,

** p<0.05,

*** p<0.01

^b^refers to discontinuation of treatment in year one of post-index period, estimated using weighted least squares

For the subgroup of individuals with major polypharmacy, the effect of taking an FDC compared to an LDC on adherence was larger in years two and three compared to the full sample (0.20, p<0.001; 0.30, p<0.001; 0.34, p<0.001). Moreover, we no longer observed any statistically significant different changes in the number of total prescriptions between the two groups, but the effect on the number of antidiabetic prescriptions remained statistically significant (-4.44, p<0.001; -3.16, p<0.001; -2.91, p<0.001). The change in the proportion of individuals with emergency visits was slightly smaller for the FDC subgroup compared to the LDC subgroup in year three (-0.14, p = 0.08). Lastly, we observed a statistically significantly smaller change in the proportion of individuals with renal failure in the FDC subgroup in years two and three (-0.19, p = 0.03, -0.17, p = 0.049).

Similarly, excluding patients with psychiatric disorders or cancer increased the size of the parameter estimate for the adherence outcome in favor of the FDC group compared to the full sample (0.22, p<0.001; 0.29, p<0.001; 0.30, p<0.001). The effect on the total number of prescriptions was smaller and less statistically significant in years two and three (-4.50, p = 0.002, -3.00, p = 0.04; -2.57, p = 0.07). In addition, we observed a weak, but statistically significant increase in the proportion of individuals with heart failure among FDC patients relative to the change experience by the LDC group in year three (0.03, p = 0.08).

### Sensitivity analyses

Calculating PDC for the LDC cohort based on the mean PDC of both substances decreased the effect size of the ATT compared to the original definition, but the results point into the same direction in all years and remained highly significant in years 2 and three (0.02, p>0.10; 0.08, p = 0.006; 0.12, p<0.001). The exclusion of individuals with pre-existing renal failure did not change the results of our main analysis apart from small deviations in the parameter estimates, and a weak, but statistically significant, effect towards a higher proportion of individuals with heart failure in the FDC cohort in year one (0.02, p = 0.09).

## Discussion

In this study we used real world data to analyze differences in medication adherence, persistence, health care utilization, therapeutic safety, morbidities, and treatment modification between two cohorts of T2D patients on metformin monotherapy who added sitagliptin to their regimen either by using an FDC or an LDC. We demonstrated that adherence increased statistically significantly in the group taking an FDC relative to the change in adherence experienced by the LDC group. Indeed, the one-year ATT was 22%, the two-year effect was 25%, and the three-year effect was 29%. Persistence was also better in patients who switched to an FDC. These results suggest that increasing the use of FDCs may represent an important strategy for improving medication adherence among T2D patients. Because adherence may influence glycaemic control, our findings could help to optimize treatment strategies for T2D [2]. Moreover, our findings indicate that the reduced dosing flexibility inherent to FDCs did not compromise safety (in terms of adverse drug events) over time. One possible explanation for this finding is that the most frequent substance combinations (i.e., 50/500 mg and 50/1000 mg) are available as both LDCs and FDCs.

Our findings are congruent with evidence from the US, where the effects of FDCs on adherence and persistence have been reported as being mainly positive [11–18]. However, compared to our findings, the effects reported in US studies are not as strong. There are several explanations for this divergence. First, there is no gold standard to measure adherence, and the strength of an effect may depend on the choice of measure. This is reflected in the results of our sensitivity analysis, in which we measured adherence to LDC as the mean of both substances’ proportion of days covered. These results were closer to those from the US, where similar calculation methods were applied. Another explanation could be differences in underlying co-payment policies and degree of regulation, because out-of-pocket spending for drugs is higher in the US compared to Germany or most other European countries [35]. Previous studies have reported a negative association between the amount of out-of-pocket payments and drug adherence among T2D patients [36–38]. If patients, in turn, can only afford some of the prescribed drugs, this would impact their adherence. Finally, differences in the effect sizes between the studies may be explained by different study designs, e.g., the absence of an active control group [11].

Since measuring short-term adherence may not be sufficient when investigating chronic diseases such as T2D, our extension of the observation period to three years means that our findings may add important information about the sustainability of the intervention. Indeed, our results suggest that the effect of taking an FDC compared to an LDC actually increases as time goes on. Moreover, we found that the change in the total number of prescriptions, as well as in the number of prescriptions attributable to T2D, was statistically significantly smaller for the FDC cohort in all three years. On the one hand, this is intuitive given that twice as many packages are required for the LDC group. On the other hand, the effect appears large when considering the statistically significantly higher adherence of the FDC cohort. We found no statistically significant different changes between the two groups with respect to the number of outpatient cases and emergency visits, although the results of two studies from the US were in favour of FDC treatment for most utilization outcomes [13, 18]. In our study, we only observed a trend towards a smaller change in the proportion of patients having emergency visits in year three (-0.14, p = 0.08) in the FDC cohort in the subgroup of individuals with polypharmacy.

Lastly, we found no substantial differences between the two groups with respect to morbidities or treatment modification. This finding might suggest an equivalence between the two regimens, despite differences in adherence. However, existing literature reports positive relationships between non-adherence and adverse diabetes outcomes [39]. Consequently, another explanation is that even a three-year observation period may not be long enough to observe differences in the studied morbidities that could result from differences in adherence. Furthermore, the individuals in both groups might reflect a relatively stable T2D population, as an inclusion criterion was that patients had not received any other antidiabetic medication throughout the study period.

Our subgroup analyses may also contribute additional insights to the literature: First, the effect of FDCs on adherence was larger among individuals who were poorly adherent. In contrast, a ceiling effect may have occurred among patients who were already adherent. Second, the effect appeared to be larger for individuals with polypharmacy, indicating that a reduction in pill burden may be more important the greater the number of medications taken. A statistically significant difference in the number of prescriptions could no longer be observed when focusing on the subgroups with a high pill burden.

Finally, the effect of FDCs on adherence was higher when T2D patients with additional severe diseases were excluded from the analysis. For example, these patients may focus more intensively on their other illnesses or a reduction in pill burden by one tablet does not have as much of an impact for them, given their more complex health care demand.

Our study has several important limitations. First, observational studies are typically associated with selection bias and baseline differences in the study population given that patients have not been randomly assigned to the different treatment groups. For instance, T2D patients taking an FDC may be more or less aware of their disease or lifestyle preferences may influence one treatment over another. To minimize this problem, we applied a two-step risk adjustment, combining EB with DiD estimation, the latter of which accounts for (time-invariant) unobserved differences between cohorts. As highlighted in S1 Table, the pre-index trends appear parallel for all outcomes, supporting the common trend hypothesis, i.e., that the LDC cohort is a suitable comparison group in our setting.

Second, because our analyses rely solely on refill patterns using administrative data (i.e., an indirect adherence measure), our outcomes measure only the theoretical availability rather than actual intake of medication. On the one hand, this could mean that we overestimated adherence if, in fact, pills were not taken. On the other hand, patients may also have been stockpiling their medication from unobserved earlier years, obtained it directly from providers (e.g., physicians dispensing drug samples to patients (rare but possible)), or from family members with the same condition, although the need for this is low in a system of universal health insurance coverage like that in Germany. In these cases, our outcome measures would underestimate adherence. In addition to this, defined daily doses as a measure of days’ supply are only the assumed average maintenance dose per day and might not reflect the individually prescribed daily dose.

Third, identifying a (multiple medication) treatment based on prescription patterns is difficult. In order to ensure that individuals are not just fine-tuning their medication plan (e.g., trying different drugs before settling on a regimen), we did not allow for any other antidiabetic prescriptions than those for metformin and sitagliptin. In accordance with previous literature, two overlaps of at least 15 days with both drugs was required for a patient to be identified as taking an LDC. The result of this approach, however, is that poorly adherent individuals may not be considered in the analyses, for example if they did not have an overlap supply of both substances despite dual therapy having been agreed upon with the physician. This would result in overestimated adherence for the LDC group.

Lastly, it should be noted that a reduction in pill burden is just one mechanism to increase adherence. Compared to other factors (e.g., patient beliefs, or quality of communication between patient and health care provider), substituting LDC through FDC could be a simple approach to improve the quality of T2D care. However, this study does not take economic implications, i.e., the cost-effectiveness, of both treatment options, into account.

## Conclusion

In conclusion, switching to an FDC achieved a significant improvement in adherence and persistence compared to switching to an LDC, suggesting that FDC therapy may provide an important strategy for increasing medication adherence among T2D patients in Germany. This finding is strengthened by not having observed any statistically significant differences between the two groups with respect to therapeutic safety or health care utilization (e.g., emergency visits) throughout the three-year study period. According to our results, the impact of FDC on adherence is strongest in (i) poorly adherent patients, (ii) patients with a high pill burden (polypharmacy), and (iii) patients who did not have a severe concomitant disease.

## Supporting information

S1 Fig Common trends. Graphical analysis of parallel trends in the outcomes between the fixed-dose and loose-dose combination cohort per quarter relative to the index date.
(PDF)

S1 Table ICD-10 coding for adverse drug events. Most frequent primary and secondary diagnosis (ICD-10) associated with adverse drug events in Germany.
(PDF)

S2 Table ICD-10 codes of disease-related morbidities. ICD-10 coding for microangiopathic and macroangiopathic complications.
(PDF)

S3 Table Differences between study cohorts. Detailed information about the percentage of individuals with comorbidities (Elixhauer groups, pharmacy-based metrics, and type 2 diabetes-specific comorbidities) in the fixed-dose combination and loose-dose combination cohorts before and after entropy balancing.
(PDF)

S4 Table Weighted mean outcomes and regression results. Mean outcomes for fixed-dose combination and weighted mean outcomes for loose-dose combination cohort in baseline (one year) and follow-up period (three years) with the respective difference-in-difference estimator, its standard error, and the p-value.
(PDF)
