# Supplementary material for: Regimen simplification and medication adherence: Fixed-dose versus loose-dose combination therapy for type 2 diabetes
Source: PLoS One. 2021 May 4;16(5):e0250993. doi: 10.1371/journal.pone.0250993 (PMC8096115; doi:10.1371/journal.pone.0250993)
Supplement: S1 Fig — Graphical analysis of parallel trends in the outcomes between the fixed-dose and loose-dose combination cohort per quarter relative to the index date. (PDF) [file pone.0250993.s001.pdf]

S1 Fig. Common trends. Graphical analysis of parallel trends in the outcomes between the fixed-dose and loose-dose combination cohort per quarter relative to the index date.

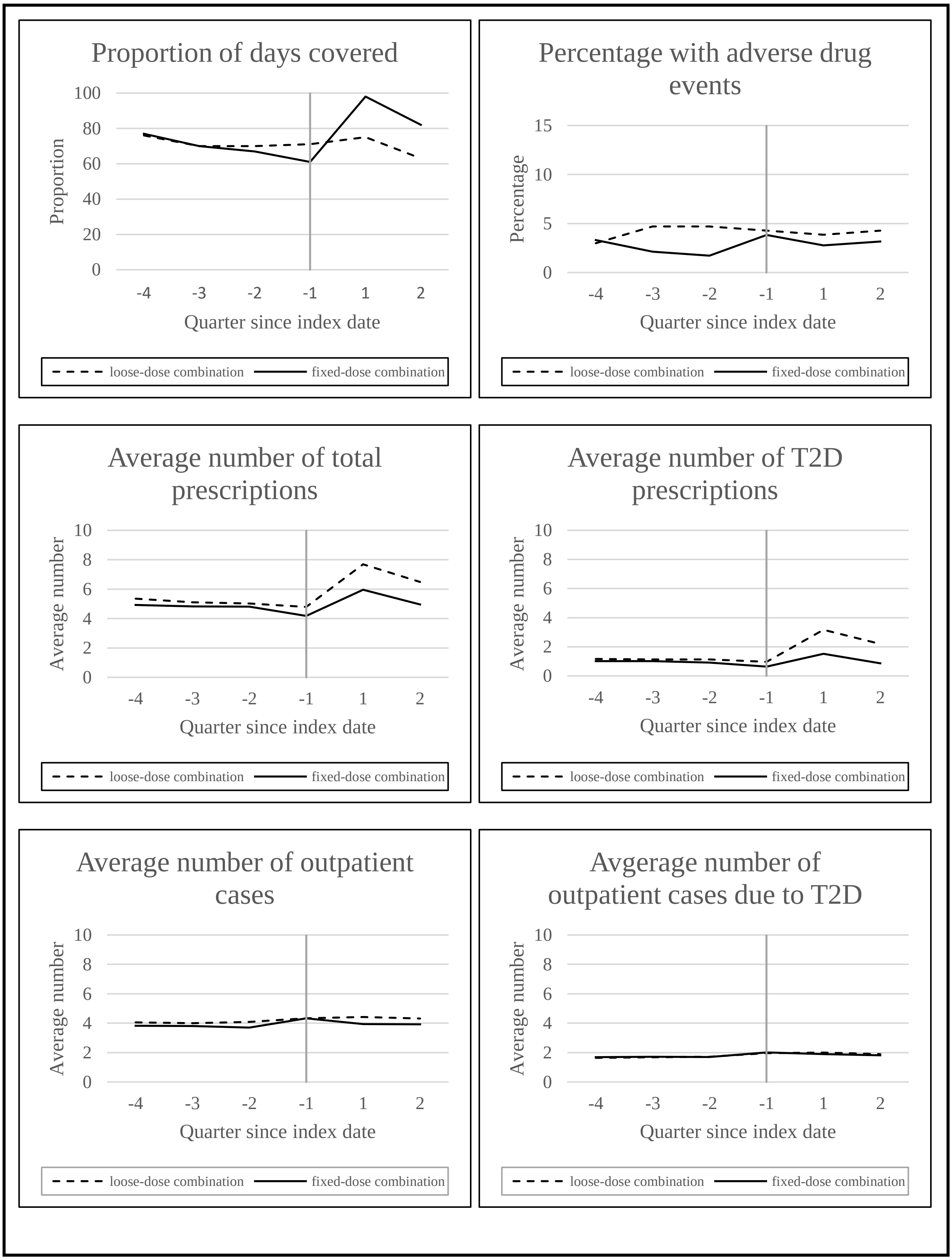

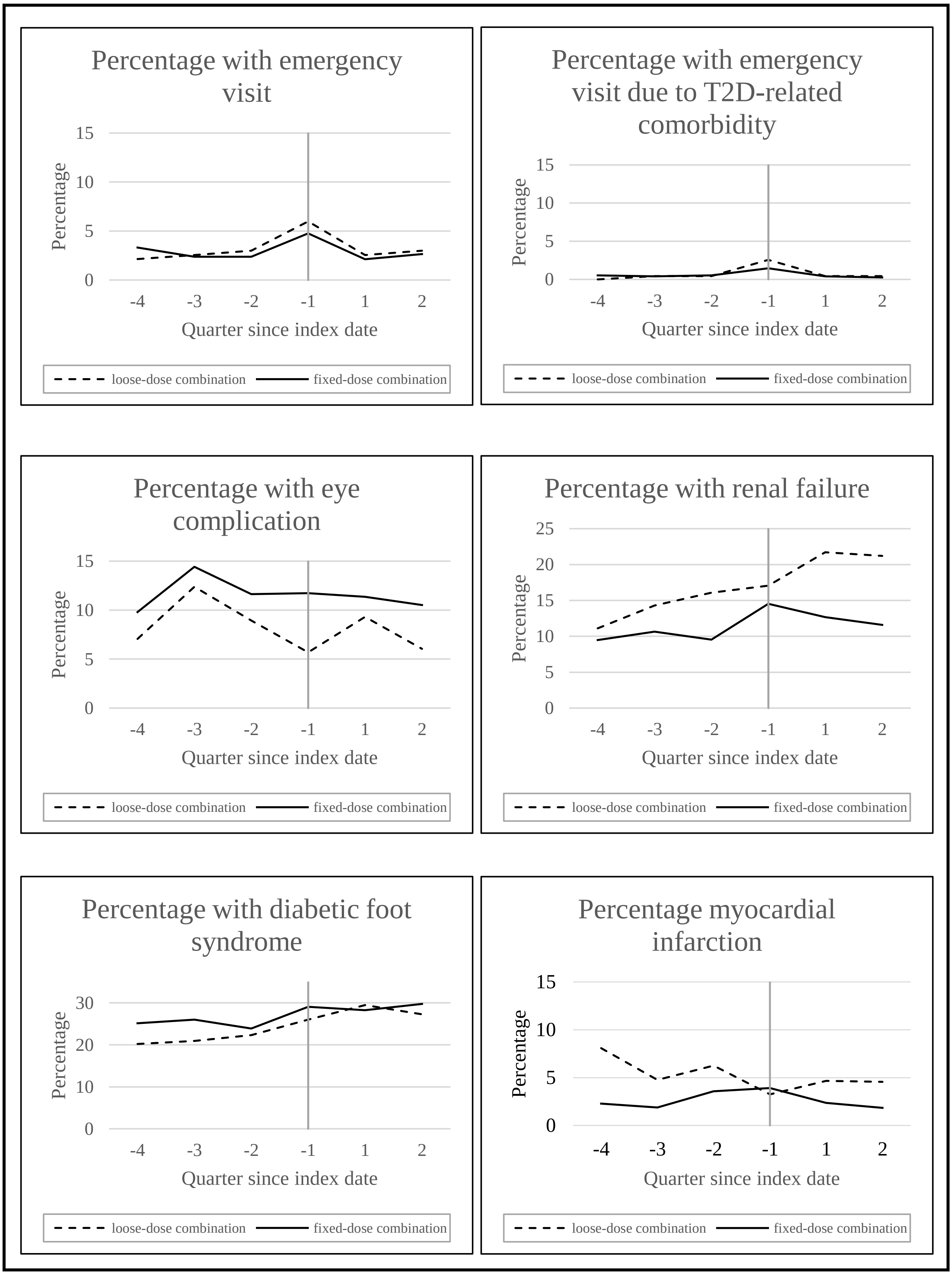

Percentage with emergency visit
Percentage
15
10
5
0
-4
-3
-2
-1
1
2
Quarter since index date
loose-dose combination
fixed-dose combination
Percentage with emergency visit due to T2D-related comorbidity
Percentage
15
10
5
0
-4
-3
-2
-1
1
2
Quarter since index date
loose-dose combination
fixed-dose combination
Percentage with eye complication
Percentage
15
10
5
0
-4
-3
-2
-1
1
2
Quarter since index date
loose-dose combination
fixed-dose combination
Percentage with renal failure
Percentage
25
20
15
10
5
0
-4
-3
-2
-1
1
2
Quarter since index date
loose-dose combination
fixed-dose combination
Percentage with diabetic foot syndrome
Percentage
30
20
10
0
-4
-3
-2
-1
1
2
Quarter since index date
loose-dose combination
fixed-dose combination
Percentage myocardial infarction
Percentage
15
10
5
0
-4
-3
-2
-1
1
2
Quarter since index date
loose-dose combination
fixed-dose combination

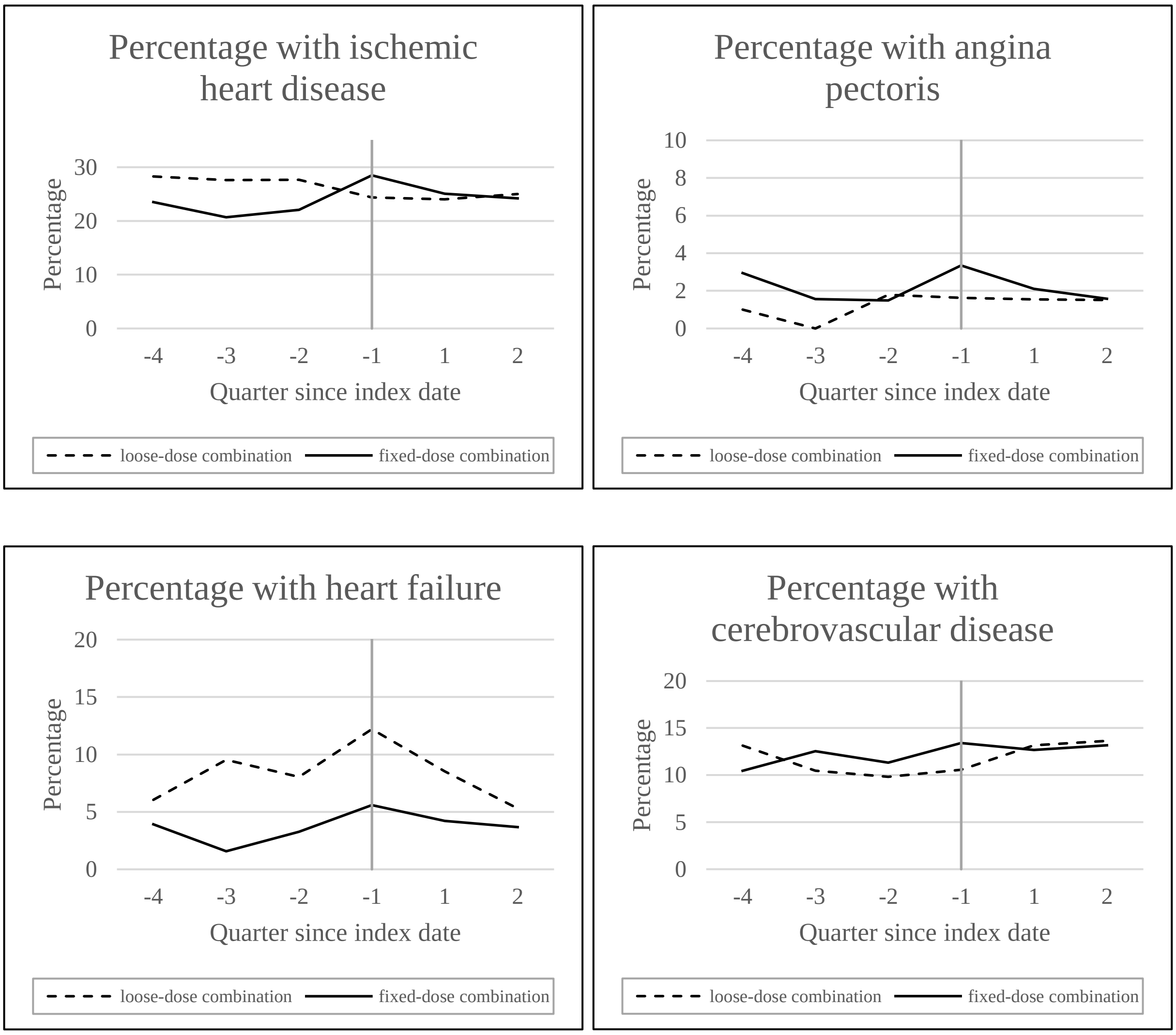

Note: Quarterly outcomes reported as entropy balancing-weighted averages or *unweighted* percentages due to better interpretability. The index date was defined as the first prescription fill of the dual therapy. The vertical line displays the last value before the index date (i.e., the index date belongs to quarter one).
