# Supplementary material for: Regimen simplification and medication adherence: Fixed-dose versus loose-dose combination therapy for type 2 diabetes
Source: PLoS One. 2021 May 4;16(5):e0250993. doi: 10.1371/journal.pone.0250993 (PMC8096115; doi:10.1371/journal.pone.0250993)
Supplement: S1 Table — Most frequent primary and secondary diagnosis (ICD-10) associated with adverse drug events in Germany. (PDF) [file pone.0250993.s002.pdf]

S1 Table. ICD-10 coding for adverse drug events. Most frequent primary and secondary diagnosis (ICD-10) associated with adverse drug events in German hospitals.

Code	Diagnosis
A04.7	Enterocolitis due to Clostridium difficile
T88.7	Unspecified adverse effect of drug or medicament
I95.2	Hypotension due to drugs
D69.0	Allergic purpura
T50.9	Poisoning by other and unspecified drugs, medicaments, and biological substances
T78.3	Adverse effect, not elsewhere classified: Angioneurotic oedema
L27.0	Generalized skin eruption due to drugs and medicaments
K52.1	Toxic gastroenteritis and colitis
D70.10	Agranulocytosis due to drugs and medicaments
T78.4	Adverse effect, not elsewhere classified: Allergy, unspecified
Y57.9	Complications due to drug or medicament, unspecified
T80.1	Vascular complications following infusion, transfusion, and therapeutic injection
D61.10	Drug-induced aplastic anaemia
D69.59	Secondary thrombocytopenia
F13.7	Residual and late-onset psychotic disorder

Based on Stausberg J, Hasford J. Identification of adverse drug events: the use of ICD-10 coded diagnoses in routine hospital data. Dtsch Arztebl Int. 2010; 107:23–9. doi: 10.3238/arztebl.2010.0023 PMID: 20140170. Tables 3, 4.
