# Supplementary material for: Regimen simplification and medication adherence: Fixed-dose versus loose-dose combination therapy for type 2 diabetes
Source: PLoS One. 2021 May 4;16(5):e0250993. doi: 10.1371/journal.pone.0250993 (PMC8096115; doi:10.1371/journal.pone.0250993)
Supplement: S2 Table — ICD-10 coding for microangiopathic and macroangiopathic complications. (PDF) [file pone.0250993.s003.pdf]

S2 Table. ICD-10 codes of disease-related morbidities. ICD-10 coding for microangiopathic and macroangiopathic complications.

Diagnoses	Codes (ICD-10)
**Microangiopathic complications**	
Eye complications	H280, H54, H431, H358, H350, H352, H360, E113
Renal failure	E112, Z49, Z992, Z940, T861, E112, N17, N18, N19, N049, N059, N083, N26, N289, T824, T861
Diabetic foot syndrome and peripheral neuropathy	E114, E115, Z894, Z895, Z896, Z897, Z898, Z899, R02, L97, L984, G590, G632, G990, G730, G62, G629, G63, M142, M146, I792, I739, I702, I74, E1174, E1175
**Macroangiopathic complications**	
Angina pectoris	I20
Myocardial infarction	I21-I23
Ischemic heart disease	I24, I25
Heart failure	I50
Cerebrovascular disease	I60-I66, I69

Based on von Ferber, Liselotte, Ingrid Köster, and Hans Hauner. "Kosten der antihyperglykämischen Behandlung des Diabetes mellitus." *Medizinische Klinik* 101.5 (2006): 384-393.
