# Supplementary material for: Regimen simplification and medication adherence: Fixed-dose versus loose-dose combination therapy for type 2 diabetes
Source: PLoS One. 2021 May 4;16(5):e0250993. doi: 10.1371/journal.pone.0250993 (PMC8096115; doi:10.1371/journal.pone.0250993)
Supplement: S3 Table — Detailed information about the percentage of individuals with comorbidities (Elixhauer groups, pharmacy-based metrics, and type 2 diabetes-specific comorbidities) in the fixed-dose combination and loose-dose combination cohorts before and after entropy balancing. (PDF) [file pone.0250993.s004.pdf]

S3 Table. Differences between study cohorts. Detailed information about the percentage of individuals with comorbidities (Elixhauser groups, pharmacy-based metrics and type 2 diabetes-specific comorbidities) in the fixed-dose combination and loose-dose combination cohorts before and after entropy balancing.

S4 Table. Differences between study cohorts. Detailed information about differences in comorbidities between cohorts.

		FDC (in %)	LDC (in %)		SMD	
			Before	After	Before	After
**Elixhauser comorbidity groups**						
1	Congestive heart failure	6.74	11.97	6.76	-0.21	0.00
2	Cardiac arrhythmias	13.23	14.96	13.24	-0.05	0.00
3	Valvular disease	5.42	5.13	5.42	0 .01	0.00
4	Pulmonary circulation disorders	1.19	2.56	1.25	-0.13	-0.01
5	Peripheral vascular disorders	11.51	11.54	11.50	0.02	0.00
6	Hypertension uncomplicated	76.59	79.91	76.63	-0.08	0.00
7	Hypertension complicated	8.33	11.97	8.35	-0.13	0.00
8	Paralysis	2.78	2.56	2.78	0.01	0.00
9	Other neurological disorders	3.44	2.99	3.44	0.02	0.00
10	Chronic pulmonary disease	13.89	16.24	13.89	-0.07	0.00
11	Diabetes uncomplicated	Population of interest, inclusion criteria				
12	Diabetes complicated	Population of interest, inclusion criteria				
13	Hypothyroidism	11.77	13.25	11.77	-0.05	0.00
14	Renal failure	6.48	6.41	6.49	0.00	0.00
15	Liver disease	20.50	24.36	20.51	-0.10	0.00
16	Peptic ulcer disease excluding bleeding	0.93	2.14	0.93	-0.13	0.00
17	AIDS/HIV	Dropped due to missing variation				
18	Lymphoma	0.53	0.43	0.53	0.01	0.00
19	Metastatic cancer	0.66	0.43	0.66	0.03	0.00
20	Solid tumor without metastasis	9.13	9.83	9.12	-0.02	0.00
21	Rheumatoid arthritis	5.56	4.70	5.59	0.04	0.00
22	Coagulopathy	1.59	3.85	1.59	-0.18	0.00
23	Obesity	36.11	34.19	36.11	0.04	0.00
24	Weight loss	0.40	0.85	0.40	-0.07	0.00
25	Fluid and electrolyte disorders	3.57	4.70	3.58	-0.06	0.00
26	Blood loss anemia	0.13	0.43	0.13	-0.08	0.00
27	Deficiency anemias	1.59	3.85	1.59	-0.18	0.00
28	Alcohol abuse	2.65	4.27	2.64	-0.10	0.00
29	Drug abuse	0.13	0.85	0.13	-0.20	0.00
30	Psychoses	0.79	2.14	0.79	-0.15	0.00
31	Depression	14.15	15.81	14.14	-0.05	0.00

S4 Table. Differences between study cohorts. Detailed information about differences in comorbidities between cohorts.

		FDC (in %)	LDC (in %)		SMD	
			Before	After	Before	After
**Pharmacy-based groups**						
1	Antiplatelet	5.03	2.14	5.02	0.13	0.00
2	Anticoagulant	7.80	10.26	7.81	-0.09	0.00
3	Epilepsy	4.23	3.85	4.23	0.02	0.00
4	Hypertension	12.04	13.68	12.03	-0.05	0.00
5	HIV	Dropped due to missing variation				
6	Tuberculosis	Dropped due to missing variation				
7	Rheumatic conditions	8.47	8.12	8.52	0.01	0.00
8	Hyperlipidemia	43.12	47.01	43.19	-0.08	0.00
9	Malignancies	Dropped due to missing variation				
10	Parkinson’s disease	1.85	1.28	1.85	0.04	0.00
11	Renal disease	Dropped due to missing variation				
12	End stage renal disease (ESRD)	Dropped due to missing variation				
13	Anti-arrhythmic	2.12	3.42	2.12	-0.09	0.00
14	Ischaemic heart disease/Angina	3.97	3.85	3.97	0.01	0.00
15	Congestive heart failure/Hypertension	69.71	72.22	69.80	-0.05	0.00
16	Diabetes	Population of interest, inclusion criteria				
17	Glaucoma	3.84	5.13	3.83	-0.07	0.00
18	Liver failure	Dropped due to missing variation				
19	Acid peptic disease	31.08	32.91	31.14	-0.04	0.00
20	Transplantation	0.26	1.28	0.27	-0.20	0.00
21	Respiratory illness, asthma	12.30	8.55	12.31	0.11	0.00
22	Thyroid disorders	17.59	15.38	17.58	0.06	0.00
23	Gout	12.83	11.97	12.82	0.03	0.00
24	Inflammatory bowel disease, chronic	0.53	0.43	0.53	0.01	0.00
25	Pain and inflammation	36.24	32.91	36.24	0.07	0.00
26	Pain	5.56	4.27	5.55	0.06	0.00
27	Depression	9.92	14.53	9.92	-0.15	0.00
28	Psychotic illness	2.12	3.42	2.12	-0.09	0.00
29	Bipolar disorders	Dropped due to missing variation				
30	Anxiety and tension	2.51	2.99	2.51	-0.03	0.00
31	Hepatitis	Dropped due to missing variation				
32	Ischemic heart disease/Hypertension	55.42	58.97	55.47	-0.07	0.00
**Disease-specific groups**						
1	Myocardial infarction	4.10	6.41	4.10	-0.12	0.00
2	Ischemic heart disease	18.39	19.66	18.44	-0.03	0.00
3	Cerebrovascular diseases	8.07	9.40	8.08	-0.05	0.00

S4 Table. Differences between study cohorts. Detailed information about differences in comorbidities between cohorts.

		FDC (in %)	LDC (in %)		SMD	
			Before	After	Before	After
4	Eye complication	16.40	15.38	16.41	0.03	0.00
5	Diabetic foot syndrome/Neuropathy	19.44	18.38	19.48	0.03	0.00
6	Renal complication	12.30	12.82	12.36	-0.02	0.00
7	Angina pectoris	3.44	1.71	3.43	0.09	0.00

FDC: fixed-dose combination, LDC: loose-dose combination, SMD: standardized mean difference
