# Supplementary material for: Regimen simplification and medication adherence: Fixed-dose versus loose-dose combination therapy for type 2 diabetes
Source: PLoS One. 2021 May 4;16(5):e0250993. doi: 10.1371/journal.pone.0250993 (PMC8096115; doi:10.1371/journal.pone.0250993)
Supplement: S4 Table — Mean outcomes for fixed-dose combination and weighted mean outcomes for loose-dose combination cohort in baseline (one year) and follow-up period (three years) with the respective difference-in-difference estimator, its standard error, and the p-value. (PDF) [file pone.0250993.s005.pdf]

S4 Table. Weighted mean outcomes and regression results. Mean outcomes for fixed-dose combination and weighted mean outcomes for loose-dose combination cohort in baseline (one year) and follow-up period (three years) with the respective difference-in-difference estimator, its standard error, and the p-value.

**S5 Table. Weighted mean outcomes and regression results.**							
	**Year 1**						
	**FDC (756)**		**LDC (234)**		**DiD estimation**		
	Baseline	Follow-up	Baseline	Follow-up	ATT	P-value	SE
Adherence and Persistence							
Proportion of days covered	0.69	0.88	0.69	0.66	0.22	<0.001	0.02
Days until discontinuation[a]	276.69	N/A	157.79	N/A	119.04	<0.001	8.31
Indicators for health care utilization							
Outpatient cases	14.07	14.06	14.80	14.96	-0.16		0.63
due to diabetes	5.98	6.15	5.78	6.01	-0.07		0.28
Pharmaceutical prescriptions	22.51	21.05	24.12	26.68	-4.02	<0.001	1.07
due to diabetes	4.23	4.14	4.83	9.32	-4.59	<0.001	0.20
Proportion with emergency visits	0.12	0.07	0.16	0.09	0.02		0.04
due to T2D comorbidities	0.03	0.01	0.03	0.01	0.01		0.02
Therapeutic safety							
Proportion with adverse drug events	0.08	0.08	0.11	0.07	0.04		0.03
Comorbidities							
Proportion with microangiopatic complications							
Eye complication	0.17	0.17	0.16	0.17	0.00		0.04
Renal failure	0.13	0.12	0.14	0.14	0.00		0.03
Diabetic foot syndrome/ Periphere neuropathy	0.21	0.21	0.21	0.19	0.02		0.04
Proportion with macroangiopatic complications							
Myocardial infarction	0.04	0.03	0.04	0.03	0.00		0.02
Ischemic heart disease	0.18	0.18	0.20	0.19	0.00		0.03
Angina pectoris	0.03	0.03	0.03	0.02	0.00		0.02
Heart failure	0.05	0.04	0.06	0.05	0.01		0.01
Cerebrovascular disease	0.08	0.09	0.09	0.09	0.00		0.02
Treatment modification[b]							
Proportion with alternative							
Oral antidiabetic prescription	N/A	0.25	N/A	0.29	0.00		0.03
Insulins	N/A	0.06	N/A	0.03	0.03	0.03	0.01

FDC: fixed-dose combination, LDC: loose-dose combination, ATT: average treatment effect on the treated represents excess outcomes attributable to FDC, SE: standard error

[a]refers to discontinuation of treatment in year one of post-index period, estimated using weighted least squares

[b]based on individuals who modified their dual theapy (N: FDC=732, LDC=229)

S5 Table. (Continued) **Weighted mean outcomes and regression results.**

	**Year 2**						
	**FDC (756)**		**LDC (234)**		**DiD estimation**		
	Baseline	Follow-up	Baseline	Follow-up	ATT	P-value	SE
Adherence and Persistence							
Proportion of days covered	0.69	0.83	0.69	0.58	0.25	<0.001	0.02
Days until discontinuation[a]	N/A	N/A	N/A	N/A	N/A		N/A
Indicators for health care utilization							
Outpatient cases	14.07	13.95	14.80	14.14	0.54		0.63
due to diabetes	5.98	6.05	5.78	5.66	0.17		0.29
Pharmaceutical prescriptions	22.51	20.20	24.12	25.29	-3.48	<0.001	1.14
due to diabetes	4.23	3.29	4.83	7.55	-3.66	<0.001	0.27
Proportion with emergency visits	0.12	0.08	0.16	0.08	0.03		0.04
due to T2D comorbidities	0.03	0.02	0.03	0.03	-0.01		0.02
Therapeutic safety							
Proportion with adverse drug events	0.08	0.08	0.11	0.08	0.03		0.03
Comorbidities							
Proportion with microangiopatic complications							
Eye complication	0.17	0.18	0.16	0.21	-0.03		0.04
Renal failure	0.13	0.18	0.14	0.19	0.01		0.04
Diabetic foot syndrome/ Periphere neuropathy	0.21	0.30	0.21	0.26	0.03		0.04
Proportion with macroangiopatic complications							
Myocardial infarction	0.04	0.03	0.04	0.02	0.02		0.02
Ischemic heart disease	0.18	0.19	0.20	0.23	-0.02		0.03
Angina pectoris	0.03	0.02	0.03	0.02	0.01		0.02
Heart failure	0.05	0.06	0.06	0.07	0.01		0.02
Cerebrovascular disease	0.08	0.10	0.09	0.11	0.00		0.02
Treatment modification[b]							
Proportion with alternative							
Oral antidiabetic prescription	N/A	0.58	N/A	0.59	0.02		0.04
Insulins	N/A	0.17	N/A	0.14	0.03		0.03

FDC: fixed-dose combination, LDC: loose-dose combination, ATT: average treatment effect on the treated represents excess outcomes attributable to FDC, SE: standard error

[a]refers to discontinuation of treatment in year one of post-index period, estimated using weighted least squares

[b]based on individuals who modified their dual theapy (N: FDC=732, LDC=229)

**S5 Table.** (Continued) **Weighted mean outcomes and regression results.**							
	**Year 3**						
	**FDC (756)**		**LDC (234)**		**DiD estimation**		
	Baseline	Follow-up	Baseline	Follow-up	ATT	P-value	SE
Adherence and Persistence							
Proportion of days covered	0.69	0.81	0.69	0.52	0.29	<0.001	0.03
Days until discontinuation[a]	N/A	N/A	N/A	N/A	N/A		N/A
Indicators for health care utilization							
Outpatient cases	14.07	13.93	14.80	14.14	0.52		0.63
due to diabetes	5.98	6.15	5.78	5.96	-0.02		0.29
Pharmaceutical prescriptions	22.51	20.22	24.12	26.70	-4.87	<0.001	1.77
due to diabetes	4.23	3.16	4.83	7.04	-3.27	<0.001	0.23
Proportion with emergency visits	0.12	0.10	0.16	0.10	0.03		0.04
due to T2D comorbidities	0.03	0.02	0.03	0.01	0.01		0.02
Therapeutic safety							
Proportion with adverse drug events	0.08	0.08	0.11	0.09	0.01		0.03
Comorbidities							
Proportion with microangiopatic complications							
Eye complication	0.17	0.16	0.16	0.23	-0.07		0.05
Renal failure	0.13	0.20	0.14	0.23	-0.02		0.04
Diabetic foot syndrome/ Periphere neuropathy	0.21	0.31	0.21	0.32	-0.02		0.05
Proportion with macroangiopatic complications							
Myocardial infarction	0.04	0.04	0.04	0.02	0.02		0.02
Ischemic heart disease	0.18	0.21	0.20	0.24	-0.01		0.04
Angina pectoris	0.03	0.03	0.03	0.04	-0.01		0.03
Heart failure	0.05	0.08	0.06	0.08	0.02		0.02
Cerebrovascular disease	0.08	0.10	0.09	0.12	-0.01		0.03
Treatment modification[b]							
Proportion with alternative							
Oral antidiabetic prescription	N/A	0.78	N/A	0.79	-0.01		0.03
Insulins	N/A	0.26	N/A	0.29	-0.03		0.04

FDC: fixed-dose combination, LDC: loose-dose combination, ATT: average treatment effect on the treated represents excess outcomes attributable to FDC, SE: standard error

[a]refers to discontinuation of treatment in year one of post-index period, estimated using weighted least squares

[b]based on individuals who modified their dual theapy (N: FDC=732, LDC=229)
